# Clinical, Cortical, Subcortical, and White Matter Features of Right Temporal Variant FTD †

**DOI:** 10.3390/brainsci14080806

**Published:** 2024-08-11

**Authors:** Jana Kleinerova, Mary Clare McKenna, Martha Finnegan, Asya Tacheva, Angela Garcia-Gallardo, Rayan Mohammed, Ee Ling Tan, Foteini Christidi, Orla Hardiman, Siobhan Hutchinson, Peter Bede

**Affiliations:** 1 Computational Neuroimaging Group, School of Medicine, Trinity College Dublin, D08 W9RT Dublin, Ireland; 2 Department of Neurology, St James’s Hospital, D08 KC95 Dublin, Ireland; 3 Department of Psychiatry, Tallaght University Hospital, D24 NR0A Dublin, Ireland

**Keywords:** ALS, FTD, MRI, biomarker, sbvFTD, neuroimaging, cognition, neuropsychology

## Abstract

The distinct clinical and radiological characteristics of right temporal variant FTD have only been recently recognized. Methods: Eight patients with right temporal variant FTD were prospectively recruited and underwent a standardised neuropsychological assessment, clinical MRI, and quantitative neuroimaging. Results: Our voxelwise grey analyses captured bilateral anterior and mesial temporal grey matter atrophy with a clear right-sided predominance. Bilateral hippocampal involvement was also observed, as well as disease burden in the right insular and opercula regions. White matter integrity alterations were also bilateral in anterior temporal and sub-insular regions with a clear right-hemispheric predominance. Extra-temporal white matter alterations have also been observed in orbitofrontal and parietal regions. Significant bilateral but right-predominant thalamus, putamen, hippocampus, and amygdala atrophy was identified based on subcortical segmentation. The clinical profile of our patients was dominated by progressive indifference, decline in motivation, loss of interest in previously cherished activities, incremental social withdrawal, difficulty recognising people, progressive language deficits, increasingly rigid routines, and repetitive behaviours. Conclusions: Right temporal variant FTD has an insidious onset and may be mistaken for depression at symptom onset. It manifests in a combination of apathy, language, and behavioural features. Quantitative MR imaging captures a characteristic bilateral but right-predominant temporal imaging signature with extra-temporal frontal and parietal involvement.

## 1. Introduction

Frontotemporal dementia (FTD) encompasses a wide spectrum of neurodegenerative disorders that may be further stratified according to clinical phenotype, genotype, or the underlying molecular pathology [1,2,3,4,5,6]. The striking clinical, radiological, genetic, and molecular heterogeneity of FTD is well recognised and clinical subtypes are defined based on unique clinical and radiological features [3,7]. On clinical grounds, language-variant and behavioural-variant phenotypes are typically distinguished first before subcategorising patients into specific categories based on detailed neuropsychological data. The overlap with Amyotrophic Lateral Sclerosis is also well recognised with a number of shared radiological and pathological features [8,9,10,11,12,13]. Individuals harbouring hexanucleotide GGGCC repeat expansions in *C9orf72,* in particular, are at risk of developing either ALS, FTD, or ALS-FTD [14,15]. ALS-FTD as a distinct entity has been recognised before the discovery of *C9orf72* repeat expansions and has been extensively studied through robust neuropsychology, post mortem, and neuroimaging studies [16,17,18].

Semantic variant primary progressive aphasia (svPPA) is an FTD phenotype that clinically manifests as anomia and impaired single-word comprehension [1,19], is radiologically defined by dominant-hemispheric anterior temporal lobe atrophy [1], and is pathologically characterised by frontotemporal lobar degeneration transactive response DNA-binding protein 43 (FTLD-TDP-43) pathology type C in the majority of cases [20]. In recent times, it has become apparent that non-dominant anterior temporal lobe atrophy presents with a distinct clinical phenotype that initially does not meet the classification criteria for svPPA [1,21]. A vast range of alternative terminology has been used to describe this entity: ‘right temporal variant FTD’, ‘right temporal semantic dementia’, ‘right temporal svPPA’, and ‘right temporal behavioural variant FTD (bvFTD)’. Clinical algorithms have been proposed to differentiate this presentation from other FTD phenotypes and other neurodegenerative disorders [22]. A recent study outlined the longitudinal clinical characteristics of this cohort, proposing dedicated classification criteria with streamlined nomenclature highlighting the main symptomatology: ‘semantic behavioural variant FTD’ (sbvFTD) [23].

The proposed classification criteria for sbvFTD require at least two core criteria: loss of empathy; difficulty identifying and naming people; rigid thought processes or complex compulsions; and at least 2 supportive criteria: object-naming difficulties, spared visuospatial functions, and spared motor speech and phonology [23]. It may be a particularly difficult diagnosis early in the course of the disease and often mistaken for psychiatric illnesses [23]. The behavioural and language manifestations later progress and overlap with other FTD phenotypes, particularly svPPA and bvFTD [24,25,26,27]. It is radiologically defined by non-dominant anterior temporal lobe atrophy with progressive bilateral orbitofrontal cortex, anterior cingulate, and contralateral anterior temporal lobe atrophy [24]. FTLD TDP-43 type C is the most commonly reported pathology [21,23,25]. While sbvFTD is increasingly recognised as a distinct phenotype, relatively few case series have been published, and it is a particularly challenging diagnosis to establish as atrophy patterns can be challenging to appreciate on standard clinical imaging. Accordingly, our objective is the detailed clinical and radiological profiling of a cohort of patients with sbvFTD using standardised clinical instruments and a standardised quantitative neuroimaging protocol.

## 2. Materials and Methods

Ethics Approval

All aspects of this project were approved by the Ethics Committee of Beaumont Hospital Dublin (REC reference: 08/90), and each participant gave informed consent prior to study enrolment.

Participants

A total of 8 participants with right temporal variant FTD and 100 healthy controls were included in this study. All patients first had standard clinical T1-weighted, FLAIR and DWI MRI imaging, and 4 patients also underwent [^18^F] FDG PET-CT imaging. A total of 7 patients and 100 healthy controls (Table 1) additionally underwent high-resolution 3D T1-weighted imaging to map patterns of grey matter atrophy and diffusion tensor imaging for quantitative white matter analyses using the same scanner and a standardised neuroimaging protocol described below. Seven patients had a standardised neuropsychological evaluation with the Edinburgh Cognitive and Behavioural ALS Screen (ECAS) [28]. Three patients had comorbid Amyotrophic Lateral Sclerosis, fulfilling the El Escorial criteria [29]. Exclusion criteria for both patients and controls included prior cerebrovascular events (strokes), prior neurosurgery, traumatic brain injury, malignancies, demyelination, and paraneoplastic syndromes. None of the healthy controls had a family history of dementia, psychiatric diagnoses, or motor neuron disease among their first- and second-degree relatives.

Neuroimaging

Neuroimaging data were acquired with a standardised protocol on a 3 Tesla Philips Achieva MR platform. The protocol included fluid-attenuated inversion recovery (FLAIR), 3D T1-weighted (T1w), and diffusion-tensor (DWI) pulse sequences. FLAIR images were acquired axially with an Inversion Recovery Turbo Spin Echo (IR-TSE) sequence with the following settings: repetition time (TR)/echo time (TE) = 11,000/125 ms, inversion time (TI) = 2800 ms, field of view (FOV) = 230 × 183 × 150 mm, voxel resolution (VR) = 0.65 × 0.87 × 4 mm. T1w images were acquired with a 3D Inversion Recovery Prepared Spoiled Gradient Recalled Echo (IR-SPGR) sequence with the following parameters: TR/TE = 8.5/3.9 ms, TI = 1060 ms, FOV of 256 × 256 × 160 mm, 160 sagittal slices with no interslice gap, flip angle (FA) = 8°, VR = 1 mm^3^, SENSE factor = 1.5. Diffusion-weighted images (DWI) were obtained with a spin-echo echo planar imaging (SE-EPI) pulse sequence to acquire DWI data with a 32-direction Stejskal–Tanner diffusion encoding scheme: TR/TE = 7639/59 ms, FOV = 245 × 245 × 150 mm, 60 axial slices with no interslice gaps, FA = 90°, VR = 2.5 mm^3^, SENSE factor = 2.5, dynamic stabilisation and spectral presaturation with inversion recovery (SPIR) fat suppression.

Morphometric analyses

Patterns of grey matter atrophy in the semantic behavioural variant frontotemporal dementia cohort were evaluated by voxel-based morphometry (VBM) in contrast to the cohort of the age- and sex-matched healthy controls. FMRIB’s FSL suite was utilised to conduct the VBM analyses. [30,31,32] Standard pre-processing pipelines were implemented with skull-removal (BET) [33], motion-correction, and tissue-type segmentation. Subsequently, grey matter partial volume images were aligned to the MNI152 standard space using affine registration. A study-specific GM template was created thereafter to which the grey matter images from each subject were non-linearly co-registered. Permutation-based non-parametric inference [34] was used for group comparisons, controlling for total intracranial volumes (TIV), sex, age, and education. TIV was calculated by linearly aligning each participant’s brain image to the MNI152 standard, and the inverse of the determinant of the affine registration matrix was calculated and multiplied by the size of the template. The threshold-free cluster enhancement (TFCE) method [35] was implemented to correct for multiple comparisons. Resulting statistical maps were visualised in FSLEYES and thresholded at *p* < 0.01 to characterise focal grey matter vulnerability patterns.

White matter analyses

Pre-processing of the raw diffusion data included eddy current corrections and skull removal before a tensor model was fitted to the data to generate maps of axial diffusivity (AD), fractional anisotropy (FA), radial diffusivity (RD), and mean diffusivity (MD). The tract-based statistics (TBSS) stream [36]of FMRIB’s FSL image analysis suite was implemented for non-linear registration and skeletonisation of each subject’s images. FA, AD, MD, and RD images were merged into a single 4D image file, and a mean FA mask was created. Permutation-based non-parametric inference was used for the voxelwise comparison of diffusivity parameters between patients with semantic behavioural variant frontotemporal dementia and healthy controls using design matrix-defined contrasts incorporating age, sex, and education as covariates. The threshold-free cluster enhancement (TFCE) method was implemented, and resulting statistical maps were thresholded at *p* < 0.0125 TFCE family-wise error (FWE).

Subcortical segmentation

The standard anatomical segmentation pipeline of the FreeSurfer image analysis suite [37] was first implemented with ‘recon-all’, which includes non-parametric non-uniform intensity normalisation, affine registration to the MNI305 atlas, intensity normalisation, skull stripping, automatic subcortical segmentation, linear volumetric registration, neck removal, tessellation of the grey matter–white matter boundary, surface smoothing, inflation to minimise metric distortion, and automated topology correction [38]. The automated subcortical segmentation pipeline of FreeSurfer relies on a probabilistic atlas [39]. Segmentation accuracy has been individually reviewed for all subjects. Estimated total intracranial volumes (eTIV) were calculated in FreeSurfer using Buckner’s approach [40] and subsequently used as a covariate in group comparisons in addition to age, sex, and education.

Data availability

Clinical, genetic, or neuroimaging data from individual patients cannot be made available due to departmental policies, but additional information on data-processing pipelines can be requested from the corresponding author.

Statistics

Group differences in age and education were examined with one-way analysis of variance, and differences in sex and handedness distributions between patients with semantic–behavioural variants and healthy controls were contrasted with chi-square (χ^2^) tests (Table 1). As described above, non-parametric permutation-based testing was utilised for voxelwise grey and white matter analyses. The design matrices included age, sex, and education as covariates and total intracranial volumes (eTIV) were included as additional covariates as for voxel-based morphometry. The resulting statistical maps were corrected for family-wise error. No statistical analyses were run on PET data.

To test the effect of group on subcortical volumes, a multivariate analysis of covariance (MANCOVA) was conducted with the volumes of individual structures as dependent variables, the study group (HC, sbvFTD) as independent factor and age, sex, education, and eTIV as covariates. In case of a significant multivariate omnibus test, post hoc univariate comparisons were considered significant at *p* < 0.05, following Bonferroni corrections for multiple comparisons to reduce Type I error. These analyses were conducted using IBM SPSS v. 29.

## 3. Results

### 3.1. Grey Matter Atrophy Patterns

**Figure 1:**
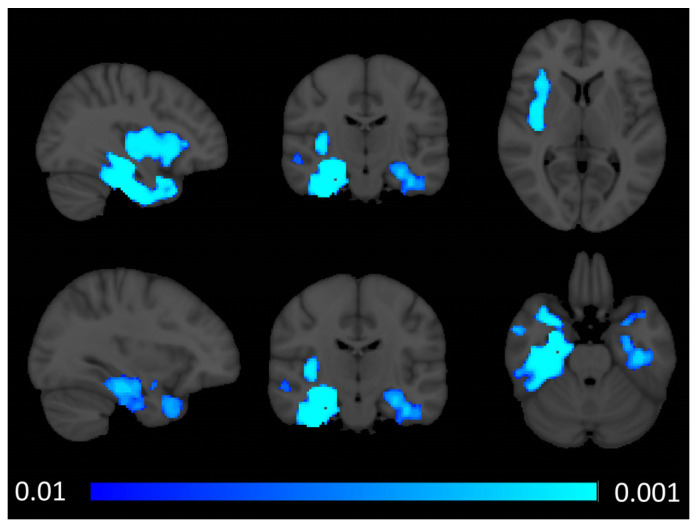
Patterns of cortical grey matter signal reduction in sbvFTD based on voxel-based morphometry outputs at *p* FWE TFCE < 0.01 corrected for age, sex, total intracranial volume, and education. MNI coordinates (x, y, z)—top row: 36, −17, 5, bottom row: −32, −17, and −26.

The demographic profiles of those included in quantitative neuroimaging analyses are summarised in Table 1. Voxel-based morphometry revealed bilateral anterior and mesial temporal atrophy with right-sided predominance. Patterns of atrophy also affected the bilateral hippocampi and the right insular and opercula region (Figure 1).

### 3.2. White Matter Patterns

**Figure 2:**
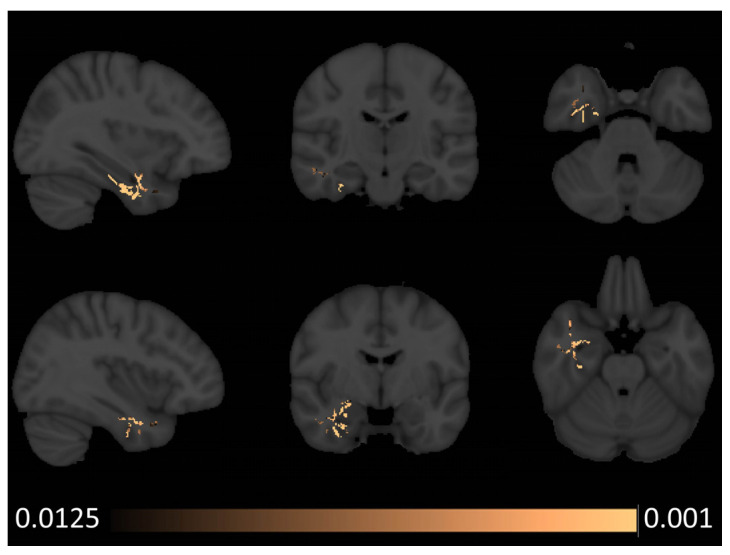
Patterns of axial diffusivity (AD) alterations in sbvFTD based on tract-based statistics outputs thresholded at *p* FWE TFCE < 0.0125 corrected for age, sex, and education. MNI coordinates (x, y, z)—top row: 34, −17, and −33; bottom row: 38, −5, and −25.

**Figure 3:**
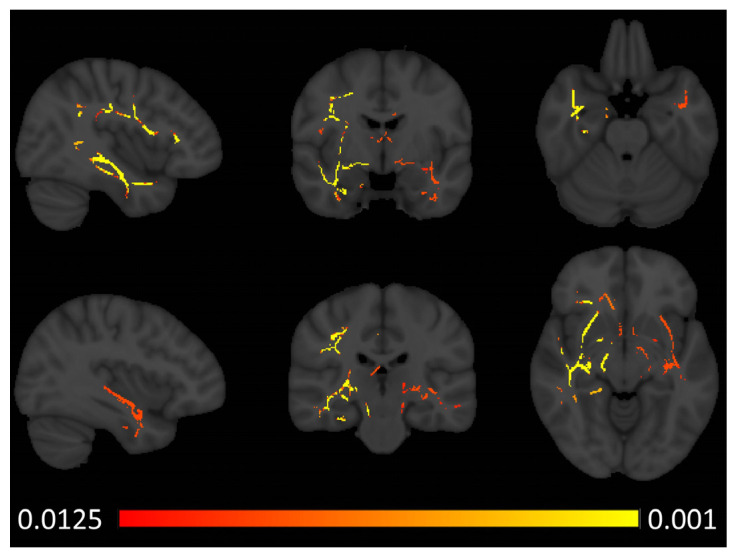
Patterns of fractional anisotropy (FA) reductions in sbvFTD based on tract-based statistics outputs thresholded at *p* FWE TFCE < 0.0125 corrected for age, sex, and education. MNI coordinates (x, y, z)—top row: 41, −5, and −24, bottom row: −36, −23, and −7.

**Figure 4:**
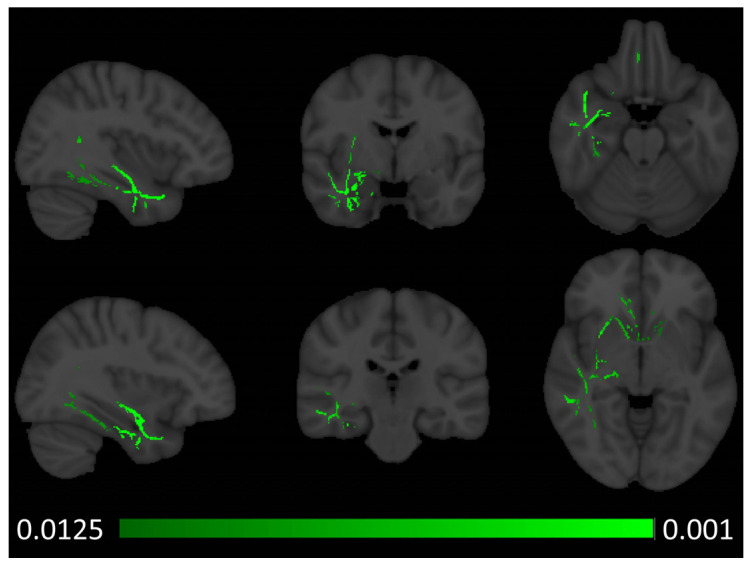
Patterns of radial diffusivity (RD) alterations in sbvFTD based on tract-based statistics outputs thresholded at *p* FWE TFCE < 0.0125 corrected for age, sex, and education. MNI coordinates (x, y, z)—top row: 37, −3, and −22, bottom row: 34, −25, and −4.

**Figure 5:**
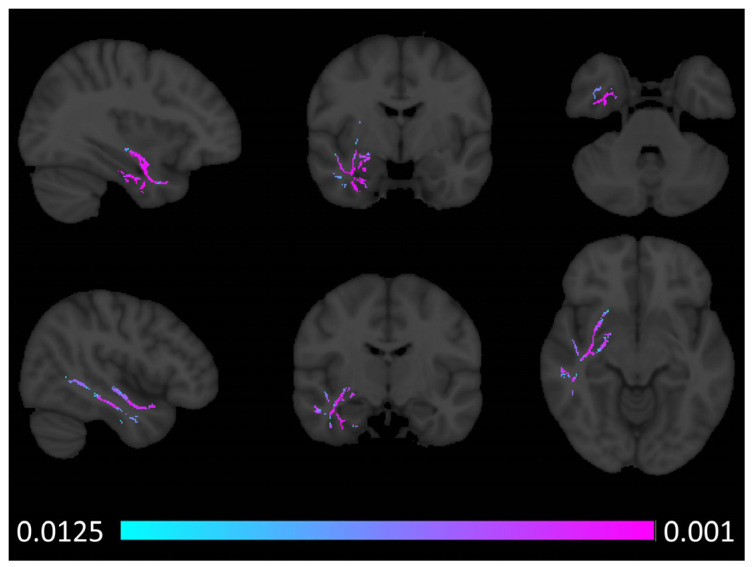
Patterns of mean diffusivity (MD) alterations in sbvFTD based on tract-based statistics outputs thresholded at *p* FWE TFCE < 0.0125 corrected for age, sex, and education. MNI coordinates (x, y, z)—top row: 34, −5, and −35, bottom row: 45, −10, and −10.

Tract-based spatial statistics captured increased axial diffusivity (AD) in the right anterior temporal lobe in patients with semantic behavioural variant frontotemporal dementia (Figure 2). Similar to the voxel-based morphometry results, fractional anisotropy (FA) reductions were noted bilaterally in both anterior temporal and sub-insular regions with a marked predominance to the right hemisphere (Figure 3). In addition to the temporal and insular white matter integrity changes, right-predominant orbitofrontal and parietal FA alterations were also noted at *p* < 0.0125 FEW-corrected. Increased radial diffusivity was noted in the right temporal lobe with a clear anterior predominance (Figure 4), but also some orbitofrontal and sub-insular involvement. Mean diffusivity increases were confined to the right anterior temporal lobe and right insular regions (Figure 5).

### 3.3. Concordance with PET

**Figure 6:**
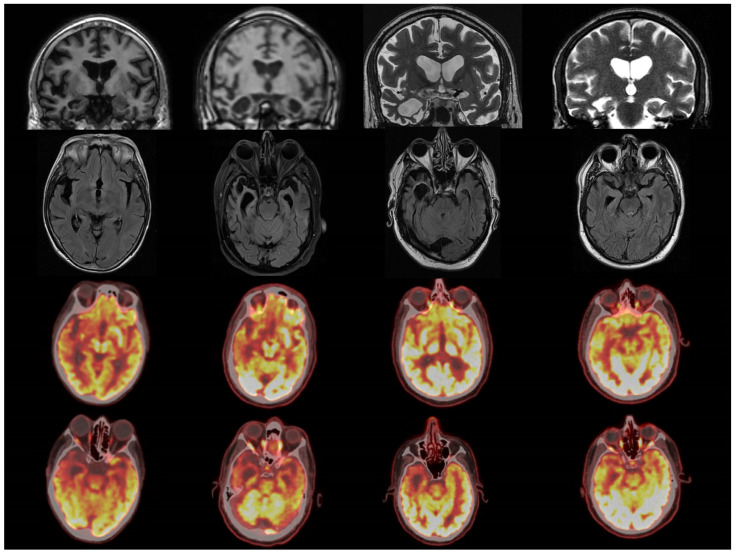
Concordance of structural changes on clinical MRIs and metabolic patterns on [^18^F] FDG PET-CT.

Four patients had additional PET imaging, which revealed a relative concordance between focal PET hypometabolism and volume loss based on visual inspection (Figure 6).

### 3.4. Subcortical Grey Matter Volume Alterations

There was a significant group effect on subcortical grey matter volumes (Pillai’s Trace = 0.684, F (14,88) = 13.608, *p* < 0.001, η^2^*p* = 0.684). Significant differences were detected in the volumes of most subcortical structures between patients with sbvFTD and controls after Bonferroni corrections for multiple comparisons: left thalamus (*p* < 0.001), left caudate (*p* = 0.014), left putamen (*p* < 0.001), left pallidum (*p* < 0.001), left hippocampus (*p* < 0.001), left amygdala (*p* < 0.001), right thalamus (*p* < 0.001), right caudate (*p* < 0.001), right putamen (*p* < 0.001), right hippocampus (*p* < 0.001), right amygdala (*p* < 0.001), and right accumbens area (*p* < 0.001). Based on η^2^*p* values, larger effect sizes were identified for most right hemispheric structures compared to the left ones. The volumetric profile of the two study groups is summarised in Table 2, reporting estimated marginal means and standard error in patients with sbvFTD and healthy controls, as well as univariate effect sizes and corrected *p*-values (Table 2).

### 3.5. Clinical Profiles

Six patients had a detailed clinical assessment with neuropsychological screening. The main findings are summarised in Table 3.

## 4. Discussion

This study highlights the core and radiological features of sbvFTD. The systematic assessment of a cohort of patients with sbvFTD in a single-centre setting and the computational analyses of MRI data acquired with a standardised radiological protocol allow the description of unifying anatomical features.

Clinical observations

Despite differences in symptom duration, our cohort exhibited relatively unifying clinical features. All cases had initial insight into their deficits. All cases presented with rigid thought processes, executive dysfunction, and varying degrees of prosopagnosia; the majority had verbal semantic loss, obsessive repetitive behaviours, and episodic memory impairment; and some also had a loss of empathy, apathy, disinhibition, alexithymia, and dietary changes. The main cognitive domains affected were executive, language, fluency, and memory. Most cases had anomia with varying levels of impaired comprehension. Surface dyslexia was also observed. All of the patients exhibited progressive indifference and a decline in motivation. The initial indifference and lack of motivation in 3 out of 8 cases were initially mistaken for depression. A loss of interest in previously cherished activities, such as reading, playing golf, and gardening, was a common theme. Incremental social withdrawal was invariably reported, which is likely to be multifactorial in the majority of cases due to loss of interest in friends and relatives, difficulty recognising people, and language deficits. Many of our patients developed an increasingly rigid routine, such as repetitively watching the same movie, going for long drives to the same location, listening to the same music, and taking the train to a specific destination to get an ice cream every day. In addition to the development of a regimented routine, increasingly rigid and obsessive behaviours were exhibited by some, such as only charging their mobile phone to exactly 100%. Difficulty recognising people, including familiar faces, is a common complaint in this cohort, and one patient explained that she mostly identifies people by their voices. Relentlessly evolving language deficits have particularly severe quality of life ramifications, impacting employment, job fulfilment, and enjoyment of social interactions. Increasing difficulty with both low- and high-frequency objects, variable levels of semantic deficits, frequent circumlocution, and perseveration increasingly affect both professional communication at work and informal communication in the community. Even with relatively preserved verbal fluency, digression into tangential anecdotes was noted in three patients. Family members of affected patients reported notable changes in the character of their loved ones. Three patients developed a preference for sweet foods, a symptom commonly observed in both FTD phenotypes and ALS [41]. One patient described a marked change in musical taste at the onset of symptoms, which the family found unusual. Inappropriate comments, reference to a passive death wish, and perseverative thoughts ruminating on previous work issues have also been observed in our cohort. A relative lack of empathy was noted in two patients when their partner became tearful during the consultation. Increasing difficulty using everyday technology, such as mobile phones and laptops, is also commonly reported. One patient became a victim of online fraud due to impaired judgment.

Radiological considerations

Our voxelwise grey analyses captured bilateral anterior and mesial temporal grey matter atrophy with a clear right-sided predominance. Bilateral hippocampal involvement was also observed, as well as disease burden in the right insular and opercula region (Figure 1). Similar to the bilateral grey matter patterns, bilateral white matter integrity alterations were noted bilaterally in anterior temporal and sub-insular regions with a clear right-hemispheric predominance. Interestingly, extra-temporal white matter alterations have also been observed in orbitofrontal (FA and RD) and parietal (FA) regions. The sensitivity profiles of the various diffusivity metrics to capture sbvFTD-associated changes differed significantly. While both FA and RD detected bilateral temporal and extra-temporal changes in orbitofrontal regions, parietal change was only detected by FA, and MD only detected right anterior temporal lobe and right insular white matter changes. FA is the most widely used diffusivity metric, which is generally considered sensitive to focal white matter integrity alterations, albeit histopathologically relatively non-specific. Traditionally, axial diffusivity is often conceptualised as an ‘axonal’ measure [42], whereas RD has been classically regarded as a myelin-related marker [43], although this interpretation of diffusivity alteration is likely to be simplistic. Notwithstanding the differences between the statistical maps generated based on the different diffusivity metrics, the integrative interpretation of the voxel-based morphometry and tract-based statistics results is that sbvFTD affects the bilateral temporal lobes, and extra-temporal frontal and parietal changes can also be readily captured. In addition to the anatomical concordance of our grey and white matter analyses, metabolic profiles on PET imaging have also been strikingly concurring (Figure 6), highlighting bilateral but right-predominant hypometabolism with varying degrees of extra-temporal involvement. The review of individual PET images reveals a degree of heterogeneity in the anatomical extent and symmetry of neurodegeneration. While some patients in our cohort exhibit relatively focal right temporal-predominant degeneration, others exhibit more widespread changes. Furthermore, while all patients had a standard clinical MRI before enrolling in this imaging study, the visual review of their original clinical T1-weighted clinical images did not permit the detection of extra-temporal atrophy. The review of their initial clinical DWI/ADC and FLAIR images was unsuitable for the detection of the underlying white matter degeneration. This highlights the importance of acquiring high-resolution 3D T1w images in the clinical setting, as these permit quantitative post hoc analyses, as demonstrated in this study. We would also advocate the inclusion of a short DTI protocol so that underlying white matter integrity changes can be mapped computationally.

Academic considerations

The clinical and radiological profiles of our cohort are largely consistent with the existing sbvFTD literature. The average age of symptom onset is typically in the early 60s [23]. It often affects highly educated individuals [23] and presents with predominantly behavioural symptoms initially, such as loss of empathy, rigid thought processes, and loss of person-specific knowledge [23,27]. It has been proposed that the terminology ‘loss of person-specific semantic knowledge’ better encapsulates the multi-modal loss of person-specific concepts—face, voice, name, or biographical information—rather than ‘prosopagnosia’, which merely refers to difficulty recognising faces [21,23]. This deficit tends to precede the loss of verbal semantic knowledge which corresponds with the anatomical progression of the disease to the contralateral anterior temporal lobe. In addition to the ‘loss of person-specific semantic knowledge’ [22], there are some early clinical features that help to distinguish sbvFTD from other FTD phenotypes, despite the considerable clinical overlap that ensues later [22,23,24]. In contrast to svPPA [1], sbvFTD presents with early behavioural rather than language impairment [25]. Compulsive behaviours tend to be driven by verbal targets (e.g., fixation on charging a phone to 100%) rather than visual targets (e.g., cleaning dishes) [25]. These behaviours include ritualistic preoccupations [22], such as getting an ice cream at the same place every day. In contrast to bvFTD [6,44], insight is initially preserved [27], episodic memory is often impaired [22,27], language dysfunction is more marked [45], dietary changes are less frequent [22], and disinhibition tends to be more subtle in sbvFTD [23]. The lateralisation of language may also influence the clinical phenotype. Most people are left-hemispheric dominant, irrespective of their handedness [46]. Indeed, in the largest case series of sbvFTD, 15% of cases were left-handed or ambidextrous [23]. From a radiological perspective, sbvFTD is classically associated with subdominant temporal lobe atrophy and hypometabolism [47]. There is progressive medial-to-lateral gradient anterior temporal lobe [22,27,48] atrophy associated with ipsilateral insular [22], hippocampal [27,48], amygdalar [25,27,48,49], and fusiform gyrus [22,48] atrophy. Subdominant temporal lobe atrophy correlates with a loss of socioemotional non-verbal semantic knowledge [23], e.g., recognising emotion [45,49,50,51,52,53], peoples’ faces [22,24], and social cues [53,54]. Focal hypometabolism correlates with psychiatric symptoms, low mood, and anxiety [55]. The disease later progresses to involve the contralateral anterior temporal lobe [22,23,25,27,48], hippocampus [27,48], amygdala [27,48], fusiform gyrus [48], bilateral anterior cingulate [24], and orbitofrontal regions [21,22,24,25,49] [22]. The degree of atrophy inversely correlates with disease duration [25]. Similar to svPPA, FTLD-TDP43 type C is the most commonly reported pathology [21,56]; however, FTLD-tau and FTLD TDP43 type A and B have also been described [56,57]. The different molecular pathologies manifest in relatively different clinical phenotypes: FTLD-TDP43 type C leads to a temporal predominant degeneration associated with prominent semantic impairment, whereas FTLD-tau and FTLD-TDP43 types A and B lead to frontal-predominant degeneration with prominent behavioural impairment [56,57].

ALS-FTD

While sbvFTD is a well-recognised clinical entity, ALS-associated sbvFTD is not typically considered as a separate phenotype. ALS-FTD has an ever-growing literature [58,59]. After a series of early descriptions of ALS cases with marked behavioural impairment [60], the identification of GGGGCC hexanucleotide repeat expansions in ALS has given considerable impetus to ALS-FTD research [61]. Imaging studies in ALS have gradually shifted their focus from primary motor regions to the evaluation of frontotemporal, cerebellar, and subcortical regions [18,59,62,63,64,65,66,67,68]. While initially, executive dysfunction, verbal fluency deficits, pseudobulbar affect, and disinhibition were considered the hallmarks of ALS-associated neuropsychological profile [12,16,60,69,70], the high incidence of comorbid language deficits, memory impairment, apathy, and deficits in social cognition were also increasingly recognised in ALS [71,72,73,74,75,76,77,78]. The practical ramifications of cognitive and behavioural impairment in ALS cannot be underestimated [79], as they impact survival, compliance with assistive devices, participation in clinical trials, and caregiver burden [80,81]. Language deficits in ALS have been previously linked to both grey and white matter degeneration [73], apathy has been linked to nucleus accumbens atrophy [82], memory impairment to hippocampal pathology [83], and amygdala [84,85] and thalamus degeneration has been implicated in multi-domain deficits [86,87], but sbvFTD is not universally recognised as a separate entity in ALS.

Subcortical involvement in ALS-FTD and FTD phenotypes

The physiology role of specific subcortical nuclei in relaying distinct cortico-cortical and cortico-basal networks is well described [88,89], and these networks have been implicated in disease propagation in ALS and ALS-FTD [90,91]. Thalamic degeneration is particularly well characterised across the spectrum of ALS-FTD phenotypes [86,92,93,94,95]. Subcortical involvement and frontotemporal dysfunction are also recognised in less common motor neuron disease phenotypes, such as primary lateral sclerosis [96,97]. Presymptomatic thalamic changes have been described in GGGGCC hexanucleotide repeat expansion carriers by several groups long before projected symptom onset [98,99,100]. However, hexanucleotide repeat expansion status is not the sole determinant for subcortical degeneration in ALS; considerable thalamic and subcortical grey matter degeneration can also be observed in *C9orf72* negative ALS cohorts [101,102]. Hippocampal degeneration also has considerable literature in both FTD [103] and ALS [83,104], and more recent studies have examined specific hippocampal subfields separately as they relay different networks and contribute to the function of specific limb circuits including Papez circuit [105,106,107]. Accumbens nucleus and amygdalar degeneration [84] are also well recognised along the ALS-FTD spectrum and have been linked to specific neurocognitive manifestations [108], but differences in left and right hemispheric pathology are seldom specifically evaluated. While the cortical profile of sbvFTD is well characterised [47,56], subcortical involvement is less well studied.

Clinical implications

As demonstrated by the clinical profile of our cohort, right temporal variant ALS-FTD/FTD may be initially mistaken for depression or psychiatric conditions, and there may be a very significant interval between symptom onset and definite diagnosis. Misdiagnoses and diagnostic delays not only cause frustration in affected families but also delay supportive interventions, genetic screening, timely resource allocation, capacity testing, etc. The wider recognition of sbvFTD will eventually lead to an earlier diagnosis of these patients, a better understanding of disease mechanisms [23], and, ultimately, the development of robust research frameworks to accurately stratify FTD phenotypes [21]. Our report also highlights the role of PET imaging in suspected cases and the limitations of visual inspection of standard clinical MRI. While in advanced disease, visual inspection of structural images may reveal ex vacuo ventricular dilation and widened sulci, these images are not suitable to appraise cortical thickness and density reductions, and visual inspection also precludes the assessment of deep subcortical grey matter volume reductions. Similarly, while FLAIR or T2w imaging would capture microvascular disease burden, degenerative changes in specific white matter tracts can only be evaluated by 3D DTI-derived or more advanced (NODDI, HARDI) white matter imaging techniques [109,110]. While until recently, quantitative neuroimaging has primarily offered group-level imaging signatures in various ALS-FTD phenotypes, with the advent of novel machine-learning [111,112,113,114,115,116] and robust z-score-based approaches [117,118,119,120], single MRI data sets can now be meaningfully interpreted [121].

Study limitations

This study is not without limitations. Despite the relative clinical and radiological homogeneity of our cohort, there were considerable differences in the symptom duration profile of our patients. A key limitation of this study stems from its cross-sectional design. While we capture and describe a unifying radiological signature, the cross-sectional analyses preclude the characterisation of the clinical and radiological evolution of disease trajectories. Only a prospective, longitudinal, multi-time point study would elucidate anatomical propagation patterns and confirm whether progressive contralateral temporal lobe atrophy ensues eventually. Finally, we do not have accompanying neuropathology data on this cohort to offer histological descriptions and TDP-43 subtyping. Owing to the small sample size of our cohort, the imaging profile of the sbvFTD group has not been systematically contrasted to other ALS-FTD phenotypes.

Future directions

Large longitudinal studies are needed with a comprehensive neuropsychological battery to assess the clinical and radiological trajectory of this entity with subsequent post mortem examination and TDP-43 subtyping. In light of the heterogeneity of temporal lobe pathology in other neurodegenerative disorders, the clinical correlates of right temporal lobe pathology should also be studied in other motor neuron diseases (PLS, HSP, and SBMA), Alzheimer’s disease, and movement disorders [122,123,124,125,126,127,128,129,130]. In the assessment of patients with suspected neurodegenerative conditions, clinical MRI protocols should routinely incorporate a 3D T1-weighted sequence and a DTI sequence to enable post hoc quantitative grey and white matter analyses, which are useful for both diagnostic and monitoring purposes.

## 5. Conclusions

Our data suggest a unifying imaging signature in sbvFTD encompassing right-predominant but bilateral temporal lobe degeneration. In addition to the striking temporal disease burden, we have also demonstrated considerable subcortical grey matter pathology, as well as insular, frontal, and parietal involvement. Due to its distinguishing clinical features, the associated diagnostic challenges and singular metabolic, diffusion, and structural signature, sbvFTD should be considered a distinct clinical phenotype along the FTLD continuum. The increasing recognition of this phenotype and increased research efforts dedicated to sbvFTD may ultimately enhance the development of consensus management protocols.

## Figures and Tables

**Table 1:** The demographic profile of patients with semantic behavioural variant FTD (sbvFTD) and healthy controls (HC) who underwent quantitative grey and white matter neuroimaging.

	Semantic–Behavioural Variant FTD (sbvFTD) n = 7	Healthy Controls (n = 100)	Group Comparisons
Age (Years—M/SD)	67.29 (4.92)	65.23 (6.52)	0.416
Sex (M/F)	4/3	56/44	0.953
Education (Years—M/SD)	14.43 (3.99)	14.92 (3.34)	0.711
Handedness (Rt/Lt)	6/1	88/12	0.858
Symptom duration (Years—M/SD)	5.86 (2.41)	n/a	n/a

Notes. Group-differences in age and education were examined with one-way analysis of variance and differences in sex and handedness distributions between patients with semantic–behavioural variant and healthy controls were contrasted with chi-square test (χ2) tests. Abbreviations: HC—healthy controls, Lt—left, M—mean, Rt—right, sbvFTD—semantic–behavioural variant FTD, SD—standard deviation.

**Table 2:** Subcortical grey matter volumetric profiles in sbvFTD and healthy controls (HC).

Subcortical Structure	Estimated Marginal Means ± S.E.		Statistics	
	HC (n = 100)	sbvFTD (n = 7)	Univariate Effect Size	*p*-Corrected
Thalamus L	7360.36 ± 81.57	5687.58 ± 314.29	η^2^*p* = 0.207	**<0.001**
Thalamus R	6916.73 ± 64.76	5180.82 ± 249.52	η^2^*p* = 0.309	**<0.001**
Caudate L	3426.86 ± 36.60	2945.77 ± 141.01	η^2^*p* = 0.097	**0.014**
Caudate R	3526.19 ± 36.89	2575.97 ± 142.12	η^2^*p* = 0.292	**<0.001**
Putamen L	4638.97 ± 48.63	3641.45 ± 187.39	η^2^*p* = 0.207	**<0.001**
Putamen R	4672.54 ± 49.51	2943.83 ± 190.77	η^2^*p* = 0.431	**<0.001**
Pallidum L	1940.44 ± 20.36	1599.42 ± 78.44	η^2^*p* = 0.148	**<0.001**
Pallidum R	1894.54 ± 25.57	1805.70 ± 98.50	η^2^*p* = 0.007	1.000
Hippocampus L	4064.14 ± 42.90	3334.91 ± 165.30	η^2^*p* = 0.152	**<0.001**
Hippocampus R	4199.83 ± 42.64	2650.79 ± 164.28	η^2^*p* = 0.451	**<0.001**
Amygdala L	1594.03 ± 20.70	1166.33 ± 79.77	η^2^*p* = 0.210	**<0.001**
Amygdala R	1833.13 ± 21.22	1047.12 ± 81.76	η^2^*p* = 0.460	**<0.001**
Accumbens area L	442.79 ± 9.33	338.15 ± 35.95	η^2^*p* = 0.072	0.084
Accumbens area R	528.35 ± 8.56	271.90 ± 32.96	η^2^*p* = 0.358	**<0.001**

Notes. Estimated marginal means ± S.E. for volumes of subcortical structures are adjusted for age, sex, education, and TIV. Post hoc univariate comparisons between groups were performed following a significant multivariate omnibus test: Pillai’s Trace = 0.684, F (14,88) = 13.608, *p* < 0.001, η^2^*p* = 0.684. Bold *p*-values are significant at *p* < 0.05 following Bonferroni corrections for multiple comparisons. Partial η^2^ effect size is interpreted as small (η^2^*p* = 0.01), medium (η^2^*p* = 0.06), or large (η^2^*p* = 0.14).

**Table 3:** The clinical profile of eight representative cases with semantic behavioural-variant FTD (sbvFTD).

	Case 1	Case 2	Case 3	Case 4	Case 5	Case 6	Case 7	Case 8
**Handedness**	Rt	Rt	Rt	Rt	Rt	Lt	Rt	Rt
**Age**	73	65	71	67	73	64	61	64
**Sex**	M	M	F	M	F	M	M	F
**Education (years)**	17	17	10	15	20	9	13	15
**Symptom Duration**	2 years	8 years	3 years	10 years	8 years	7 years	6 years	7 years
**Presenting symptoms** Prosopagnosia Rigid thought process Executive deficits Obs. Rep. behaviours Verbal semantic loss Ep. Mem. impairment Disinhibition Loss of empathy Apathy Dietary changes Alexithymia	✓ ✓ ✓ ✓ - - ✓ ✓ ✓ ✓ ✓	✓ - ✓ - ✓ ✓ - ✓ - - ✓	✓ ✓ ✓ ✓ - ✓ - ✓ ✓ - -	✓ ✓ ✓ ✓ ✓ ✓ ✓ - - - ✓	✓ ✓ ✓ ✓ ✓ ✓ ✓ - - - -	✓ ✓ ✓ - ✓ ✓ - ✓ ✓ - -	✓ ✓ ✓ ✓ ✓ - ✓ - ✓ - ✓	- ✓ ✓ - ✓ - ✓ - ✓ ✓ -
**Comorbid dx.**	-	Hypertension	ALS	-	-	-	ALS	ALS
**ACE-III**	97/100	89/100	63/100	41/100	91/100	88/100	61/100	93/100
**ECAS**	120/136	95/136	85/136		109/136	92/136	81/136	112/136
**BNT**	25/30	24/30	21/30	-	14/30	21/30	17/30	19/30
**Main domains** **Affected on testing**	Executive	Executive	Executive	Executive	Executive	Executive	Executive	Executive
	Language	Language	Language	Language	Language	Language	Language	Language
	Memory	Memory	Memory	Memory	Memory	Memory	Memory	Memory
	-	Fluency	-	Fluency	Fluency	Fluency	Fluency	Fluency
	Attention	-	-	-	-	Attention	-	-
**CSF**	Not AD-compatible	-	-	-	Not AD-compatible	Not AD-compatible	-	-
**AB42** **(591–997 pg/mL)**	835	-	-	-	959.6	722.2	-	-
**Total Tau** **(135–345 pg/mL)**	602.7	-	-	-	249	302	-	-
**P-Tau** **(35.0–64.0 pg/mL)**	116.5	-	-	-	46	67.3	-	-
**Neuroimaging** Clinical MRI [18F] FDG PET-CT Quantitative 3D MRI	✓ ✓ ✓	✓ - ✓	✓ - ✓	✓ ✓ -	✓ ✓ ✓	✓ ✓ ✓	✓ - ✓	✓ - ✓

**Notes**: **ACE**—The Addenbrooke’s Cognitive Examination, **BNT**—The Boston Naming Test, **CSF**—cerebrospinal fluid, **Dx.**—diagnosis, **ECAS**—Edinburgh Cognitive and Behavioural ALS Screen, **Ep. Mem. impairment**—episodic memory impairment, **FDG PET-CT**—fluorodeoxyglucose positron emission tomography, **Lt**—left, **M**—mean, **Obs. Rep. behaviour**—obsessive repetitive behaviours, **Rt**—right.

## Data Availability

Clinical, genetic, or neuroimaging data from individual patients cannot be made available due to departmental policies. Additional information on data-processing pipelines can be requested from the corresponding author.

## Glossary

AD: Axial Diffusivity, ALS: amyotrophic lateral sclerosis, ALSod: ALS online database, ANOVA: analysis of variance (ANOVA), ASO: antisense oligonucleotide, BOLD: blood-oxygen-level-dependent (BOLD) signal, C9+: ALS patients with GGGGCC hexanucleotide repeat expansion in *C9orf72*, C9-: ALS patients without GGGGCC hexanucleotide repeat expansion in *C9orf72*, C9orf72: chromosome 9 open reading frame 72, CC: corpus callosum, CT: cortical thickness, CST: corticospinal tract, DTI: diffusion tensor imaging, DWI: diffusion-weighted imaging, EPI: echo-planar imaging, FA: fractional anisotropy, FC: functional connectivity, fMRI: functional MRI, FLAIR: fluid-attenuated inversion recovery, FOV: field of view, FSL: FMRIB’s Software Library, FTD: frontotemporal dementia, FTLD: frontotemporal lobar degeneration, FWE: familywise error, GM: grey matter, HARDI: High-Angular-Resolution Diffusion Imaging, HC: healthy control, IR-SPGR: inversion recovery prepared spoiled gradient recalled echo, LH: left hemisphere, Lt: Llft, LMN: lower motor neuron, M1: primary motor cortex, MANCOVA: multivariate analysis of covariance, ML: machine learning, MND: motor neuron disease, MNI: Montreal Neurological Institute, MNI152: Montreal Neurological Institute 152 standard space, MRI: magnetic resonance imaging, MRS: MR spectroscopy, NISALS: Neuroimaging Society in ALS, NIV: non-invasive ventilation, NODDI: neurite orientation dispersion and density imaging, PBA: pseudobulbar affect, PCR: polymerase chain reaction, PD: Parkinson’s disease, PMC: primary motor cortex, QC: quality control, RH: right hemisphere, Rt: right, RD: radial diffusivity, ROI: region of interest, rs-fMRI: resting-state functional MRI, SC: structural connectivity, SD: standard deviation, SE-EPI: spin echo planar imaging, SENSE: sensitivity encoding, SPIR: spectral presaturation with inversion recovery, T: Tesla, T1w: T1-weighted imaging, TCV: total cerebellar volume, TDI: Track Density Imaging, TE: echo time, TI: inversion time, TIV: total intracranial volume, TR: repetition time, UMN: upper motor neuron, VR: voxel resolution, WM: white matter.
